# Integrated PET/MRI for the evaluation of gray matter atrophy and metabolic changes in idiopathic Parkinson’s disease

**DOI:** 10.3389/fnagi.2026.1757469

**Published:** 2026-07-07

**Authors:** Yanxia Yu, Yujie Bai, Shasha Xu, Ruifang Wang, Zhuo Wang, Ruihua Wang, Jingliang Cheng

**Affiliations:** 1 Department of Nuclear Medicine, First Affiliated Hospital of Zhengzhou University, Zhengzhou, China; 2 Key Laboratory of Molecular Imaging Medicine of Henan Province, Zhengzhou, China; 3 Department of Magnetic Resonance Imaging, First Affiliated Hospital of Zhengzhou University, Zhengzhou, China

**Keywords:** ^18^F-flurodeoxyglucose, gray matter, idiopathic Parkinson’s disease, magnetic resonance imaging, *Z*-score

## Abstract

**Background:**

The underlying mechanisms linking structural and metabolic alterations of idiopathic Parkinson’s disease (IPD) remain poorly understood. Utilizing integrated PET/MR for its precise spatial correlation, this study investigated the temporal relationship between gray matter (GM) atrophy and reduced FDG SUVR (RFS) across IPD stages to provide insights for early diagnosis.

**Methods:**

This prospective study recruited 90 IPD patients stratified by Hoehn-Yahr (H-Y) stage into mild, moderate, and severe groups, alongside 30 age-matched healthy controls (HC). All participants underwent integrated ^18^F-FDG PET/MR imaging. SPM was used to extract the GM volume (VGM) and standardized uptake value ratio for each brain region. Group differences were analyzed with one-way ANOVA and Bonferroni tests. Spearman correlation analysis was utilized to evaluate the associations of both VGM and metabolic changes with H-Y staging. Individual *Z*-scores for VGM and metabolic alterations were calculated, and their relationship was examined using Spearman/Pearson correlation, followed by paired t-tests to compare their differences.

**Result:**

With disease progression, GM atrophy and RFS in IPD patients gradually extend from focal to widespread patterns. A characteristic pattern of cerebral hypermetabolism was observed in IPD. H-Y stage demonstrated a significant inverse correlation with the extent of both brain GM atrophy (affecting 82 out of 90 regions) and RFS (affecting 20 regions). Conversely, it was positively correlated with hypermetabolism in regions including the sensorimotor cortex, amygdala, and globus pallidus. Moderate correlations between atrophy and RFS were confined to the left inferior parietal lobule (*r* = 0.619, *p* < 0.001) and superior temporal gyrus of the temporal pole (*r* = 0.612, *p* < 0.001) only in severe group. In early stages, the number of regions where RFS predominated over atrophy (57 regions) substantially exceeded those where atrophy was more severe (1 region). In late stages, the number of regions with more pronounced atrophy than RFS (25 regions) surpassed that of regions where RFS was dominant (12 regions).

**Conclusion:**

Widespread RFS predominates during early stages, whereas structural atrophy gradually becomes the dominant imaging feature as the disease advances. These alterations are significantly correlated with disease severity, underscoring the potential of multimodal imaging biomarkers for the early diagnosis of IPD and monitor its progression.

## Introduction

Idiopathic Parkinson’s disease (IPD) is an age-related neurodegenerative disorder, accounting for 70–80% of Parkinsonian syndromes and affecting approximately 3% of individuals aged 65 years or older (Lauretani et al., 2021; Barber et al., 2025; Kempster, 2025). With global population aging, the incidence and prevalence of IPD continue to rise (Barber et al., 2025). In neuroimaging, magnetic resonance imaging (MRI), amyloid imaging, and ^18^F-fluorodeoxyglucose (FDG) positron emission tomography (PET) have been successfully integrated into diagnostic criteria for Alzheimer’s disease (AD) (Dubois et al., 2014). Disease-specific imaging biomarkers have also been proposed for other neurodegenerative disorders, such as frontotemporal degeneration and primary progressive aphasia (Chen and Kantarci, 2020; Chollet and Payoux, 2022; Roytman et al., 2022). However, the application of imaging biomarkers in IPD diagnosis remains limited. Currently, the only recognized cerebral biomarker is the detection of normal presynaptic dopaminergic function via molecular imaging, primarily used to exclude IPD diagnosis (Nicastro et al., 2023; Yang et al., 2025).

Structural MRI and FDG-PET metabolic imaging, as multimodal imaging biomarkers, have important value in the differential diagnosis between IPD and Parkinson-plus syndromes (Sampedro et al., 2019; Sarasso et al., 2021; Seiffert et al., 2022; Zhao et al., 2024; Han et al., 2025). However, due to the lack of unified imaging diagnostic criteria, they have not yet been formally incorporated into the diagnostic criteria for IPD. Currently, studies on the structure-metabolism correlation remain notably insufficient (Gonzalez-Redondo et al., 2014; Albrecht et al., 2019; Liu et al., 2020). Clarifying the temporal sequence of structural changes and FDG metabolism can provide sensitive imaging biomarkers for the early diagnosis of IPD, while tracking the dynamic evolution of the differences between the two can help objectively assess disease progression and treatment response. Based on existing evidence, we hypothesize that metabolic changes may precede structural atrophy, serving as a more sensitive early imaging biomarker. Current studies mostly use separate acquisitions with independent devices, with variations in analytical methods, temporal and spatial acquisition parameters. This leads to high heterogeneity in the results and makes it difficult to accurately determine the true temporal relationship between structural changes and FDG metabolism. Integrated PET/MR technology enables simultaneous PET and MR image acquisition, allowing precise registration of metabolic function and anatomical structure, thereby effectively mitigating issues caused by temporal and spatial mismatches in conventional multimodal imaging studies. Utilizing integrated PET/MR, this study aims to investigate the temporal sequence and interrelationship between gray matter (GM) atrophy and reduced FDG SUVR (RFS) across different stages of IPD, with the goal of providing imaging biomarkers for early diagnosis of the disease.

## Methods

### Participants

Ninety IPD patients diagnosed. at the First Affiliated Hospital of Zhengzhou University (August 2022–August 2024) were stratified by disease severity [Hoehn and Yahr (H-Y) staging]: mild IPD (IPD-L, H-Y 1, *n* = 30), moderate IPD (IPD-M, H-Y 2, *n* = 30), and severe IPD (IPD-S, H-Y 3–5, *n* = 30). Thirty age- and sex-matched healthy controls (HC, Han Chinese, non-consanguineous) were recruited. All participants underwent ^18^F-FDG PET/MR imaging and comprehensive clinical evaluation (age, sex, disease duration, symptoms, and signs). To avoid the confounding effects of anti-Parkinsonian medications on cerebral ^18^F-FDG metabolic patterns, all patients were untreated or had discontinued their anti-Parkinsonian drugs for at least 12 h prior to PET/MR imaging (Feigin et al., 2001; Aljuaid et al., 2019). IPD diagnosis based on the 2015 International Parkinson and Movement Disorder Society Clinical Diagnostic Criteria (Postuma et al., 2015). This study was conducted in accordance with the Declaration of Helsinki and was approved by the Ethics Committee of the First Affiliated Hospital of Zhengzhou University. Informed consent was provided by all individual participants. Exclusion criteria were as follows: (1) secondary parkinsonism and atypical parkinsonian syndrome; (2) a history of organic brain disease, mental disorders, or other conditions that could affect brain structure and function; (3) a history of tumors.

### PET/MR imaging acquisition

^18^F-FDG was synthesized using the fully automated chemical method with the HM-20 cyclotron accelerator and CFN-100 synthesis module from Sumitomo Corporation, Japan. The radiopharmaceutical underwent automatic clinical quality control to ensure radiochemical purity of ≥98%. Patients fasted for 6 to 8 h, blood glucose levels were measured and maintained below 8.3 mmol/L. ^18^F-FDG was injected intravenously through the elbow vein at a dose of 3.70–4.44 MBq/kg. Before and after the injection, patients should remain calm, rest comfortably in a light-shielded room, and minimize sensory stimuli by using earplugs and closing their eyes.

Imaging of brain metabolism was performed using the third-generation integrated PET/MR (Biograph mMR) from Siemens, Germany. Before the examination, metallic items were removed from the patients, and their bladder was emptied. Patients were positioned in a supine posture. A 16-channel head-and-neck specialized coil was utilized for imaging, covering the range from the vertex of the skull to the chin. Utilize the same-bed synchronous acquisition mode to collect PET and MR signals, ensuring optimal temporal and anatomical registration between PET and MR data. Parameters for PET acquisition included a matrix of 344 × 344, 4 iterations, 21 subsets, a full-width at half-maximum (FWHM) of 3 mm, Gaussian reconstruction type, and image acquisition in List mode. MR sequence parameters for Sagittal 3D T1-weighted imaging (T1WI) MPRAGE (Magnetization Prepared Rapid Gradient Echo): TR 2400 ms, TE 2.26 ms, FOV 256 × 256 mm, Matrix 256 × 256, slice thickness 1 mm with 0.5 mm interslice gap.

### Image preprocessing

The preprocessing of images was performed on the MATLAB R2017a platform using the SPM12 software.^1^ After setting the spatial coordinate origin of the converted niftii file, the T1-MPRAGE image was further normalized to a standard brain structural template using the SHOOTING registration algorithm in the Cat12 toolbox to achieve high-precision nonlinear spatial normalization based on tissue probability maps (TPM) (Gaser et al., 2024), resulting in MR images with standardized anatomical spatial coordinates, and then proceed with segmentation. The quality report of the segmented image should achieve grade A or grade B. Finally, the gray matter volume (VGM) of each brain region was extracted based on the Automated Anatomical Labeling (AAL) atlas.

**Figure 1:**
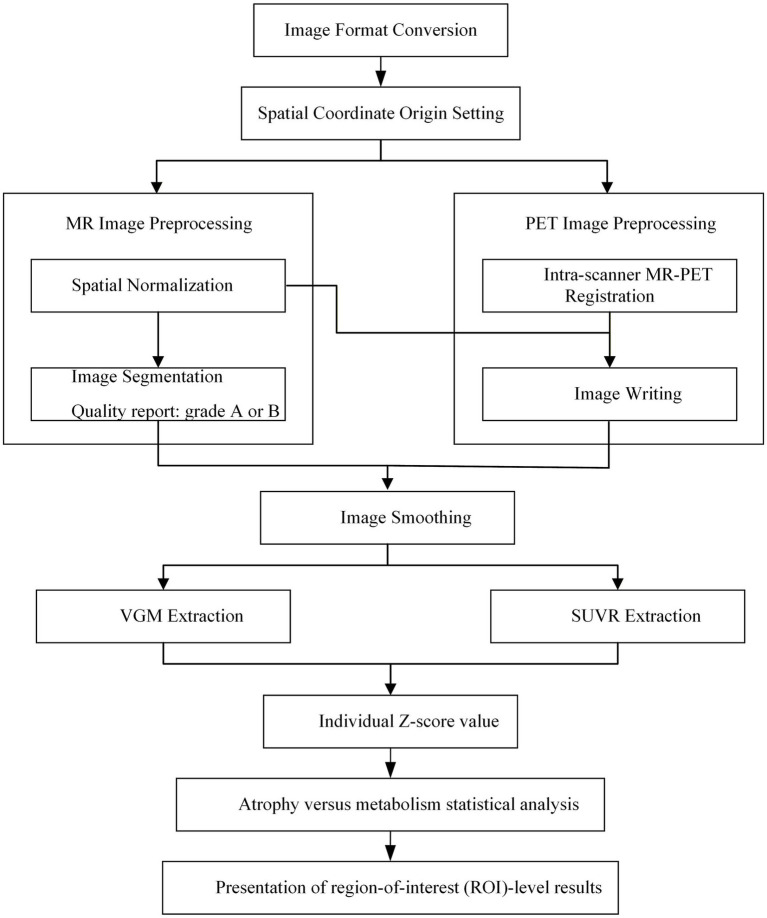
Image preprocessing flowchart.

The PET images were registered to the T1-MPRAGE image acquired from the same scanner. The T1-MPRAGE image was spatially normalized to a standard brain structural template using TPM to obtain an MR image with standard anatomical coordinates. This normalized MR image was then written into the PET image after MR registration. The voxel sizes of both MR and PET images were set to 1.5 mm × 1.5 mm × 1.5 mm, with smoothing using an 8 mm × 8 mm × 8 mm FWHM Gaussian kernel on the normalized images (Breen et al., 2016; Tsutsui et al., 2018; Stokelj et al., 2025). Furthermore, the standardized uptake value ratio (SUVR) for each brain region was calculated using the whole-brain mean as the reference region (Du et al., 2024; Lindstrom et al., 2020). The image preprocessing flowchart is shown in Figure 1.

### Statistical analysis

Statistical analyses were conducted using IBM SPSS Statistics 27.0. One-way analysis of variance (ANOVA) was employed to compare differences in VGM and SUVR metrics among the four groups: HC, IPD-L, IPD-M, and IPD-S. If significant differences were identified, post-hoc pairwise comparisons were further conducted using the Bonferroni method. Spearman rank correlation analysis was utilized to examine the correlations of VGM and SUVR with H-Y staging. To quantify the severity of brain atrophy and RFS, this study calculated *Z*-scores according to the method of Gonzalez-Redondo et al. (2014) using the formula *Z*-score = (−1) × (individual measurement − mean value of the HC group)/standard deviation of the HC group. Under this definition, a positive *Z*-score indicates more severe VGM atrophy and lower metabolic levels indicated by the SUVR in individual brain regions relative to HC, enabling direct comparative analysis of structural and metabolic abnormalities using the following methods. The normality of the MR and PET *Z*-score distributions was assessed using the Shapiro–Wilk test. For variables that followed a normal distribution (*p* > 0.05), Pearson correlation coefficient was used to analyze the correlation between MR and PET *Z*-scores; otherwise, Spearman’s rank correlation coefficient was applied. Paired t-test was used to compare the differences between the two sets of *Z*-scores. A threshold of *p* < 0.05 was considered statistically significant for all analyses.

## Results

### Clinical and demographic characteristics

**Figure 2:**
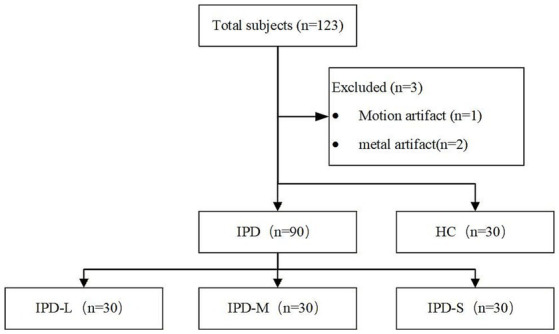
Flowchart of IPD patients enrollment.

An overview of the case selection process is shown in Figure 2. Detailed demographic and clinical characteristics are presented in Table 1.

**Table 1:** Demographic and clinical characteristics.

Clinical features	HC	IPD	IPD-L	IPD-M	IPD-S	*p* value
Participants	30	90	30	30	30	—
Sex: Male/Female	15/15	45/45	14/16	12/18	19/11	0.796^a^; 0.436^b^; 0.297^c^; 0.602^d^; 0.194^e^; 0.071^f^
Age (years)	55.77 ± 8.40	62.33 ± 10.58	55.8 ± 7.09	64.33 ± 9.17	66.87 ± 11.82	0.989^a^; <0.001^bcde*^; 0.293^f^
Disease Duration (months)	—	37.96 ± 31.81	15.8 ± 8.93	31.47 ± 28.19	66.6 ± 28.81	<0.001^de*^; 0.307^f^
Education (years)	11.83 ± 3.01	11.36 ± 3.74	13.47 ± 2.53	10.5 ± 3.19	10.1 ± 4.39	0.061^a^; 0.13^b^; 0.047^c*^; <0.001^de*^; 0.645^f^

Data are given as mean ± standard deviation. *Statistically significant differences (*p* < 0.05). ^a^Comparison among HC and IPD-L patients. ^b^Comparison among HC and IPD-M patients. ^c^Comparison among HC and IPD-S patients. ^d^Comparison among IPD-L and IPD-M patients. ^e^Comparison among IPD-L and IPD-S patients. ^f^Comparison among IPD-M and IPD-S patients.

### Structural and functional variations across IPD stages

**Figure 3:**
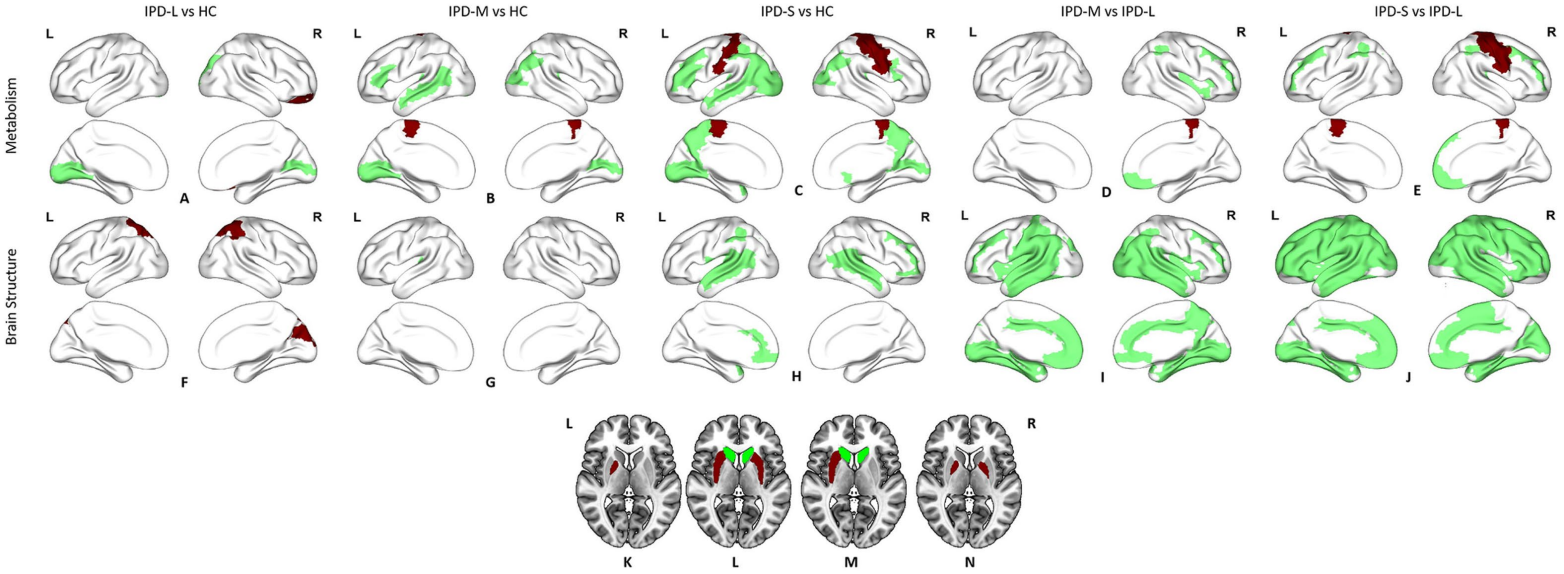
**(A–E)** Cerebral cortical FDG-PET metabolic alterations across IPD stages. **(F–J)** Cerebral cortical structural alterations across IPD stages. **(K)** Basal ganglia nuclei structural alterations in patient groups (IPD-M, IPD-S) versus HC. **(L)** Basal ganglia nuclei metabolic alterations in IPD-M versus HC. **(M)** Basal ganglia nuclei metabolic alterations in IPD-S versus HC. **(N)** Basal ganglia nuclei metabolic alterations in IPD-M versus IPD-L patients. Red indicates structural enlargement or hypermetabolism, and green indicates structural atrophy or RFS. L, left; R, right.

With disease progression, GM atrophy and RFS in IPD patients gradually extend from focal to widespread patterns. A characteristic pattern of cerebral hypermetabolism was also observed in IPD. The results of alterations in brain structure and metabolism are presented in Figure 3, with detailed data provided in Supplementary Table S1.

### Correlation analysis between H-Y staging and brain structure/metabolism

**Figure 4:**
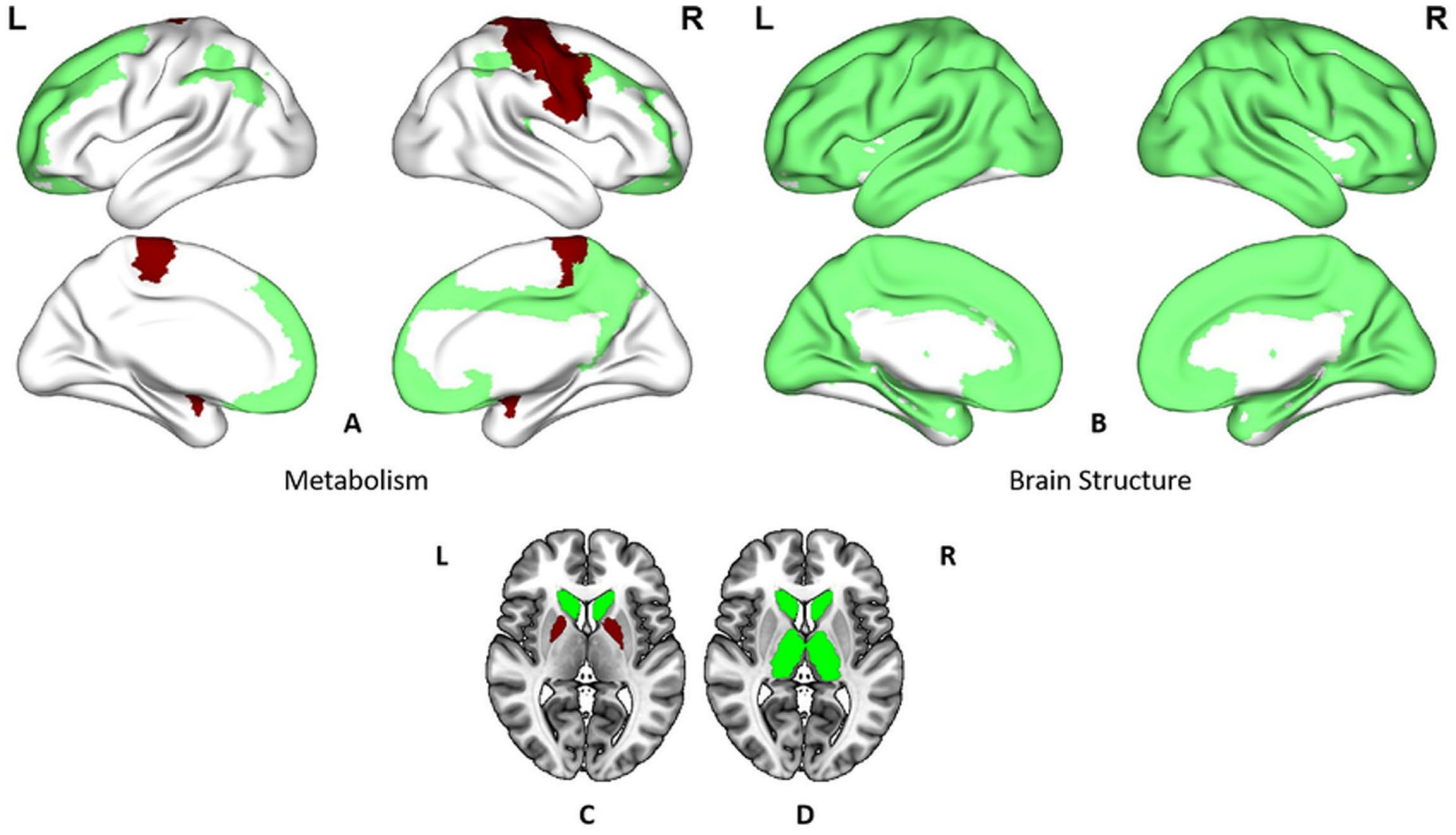
**(A)** Correlation of H-Y stage with cerebral cortical metabolism. **(B)** Correlation of H-Y stage with cerebral cortical structure. **(C)** Correlation of H-Y stage with basal ganglia nuclei metabolism. **(D)** Correlation of H-Y stage with basal ganglia nuclei structure. Red indicates structural enlargement or hypermetabolism, and green indicates structural atrophy or RFS. L, left; R, right.

H-Y stage demonstrated a significant inverse correlation with the extent of both brain GM atrophy (affecting 82 out of 90 regions) and RFS (affecting 20 regions). Conversely, it was positively correlated with hypermetabolism in regions including the sensorimotor cortex, amygdala, and globus pallidus. The results of the correlation analysis between H-Y staging and brain structure/metabolism are presented in Figure 4, with detailed data provided in Supplementary Table S2.

### Correlation between cerebral atrophy and RFS

A moderate correlation between cerebral atrophy and RFS was observed exclusively in patients with IPD-S, specifically in the left inferior parietal lobule (*r* = 0.619, *p* < 0.001) and temporal pole of superior temporal gyrus (*r* = 0.612, *p* < 0.001). No significant or only weak correlations were detected in other brain regions. Normality testing using the Shapiro–Wilk test confirmed that the MR and PET *Z*-scores for the left inferior parietal lobule (*p* = 0.152 and *p* = 0.285, respectively) and for the temporal pole of the superior temporal gyrus (*p* = 0.991 and *p* = 0.735, respectively) followed a normal distribution.

### Comparison between cerebral atrophy and RFS

**Figure 5:**
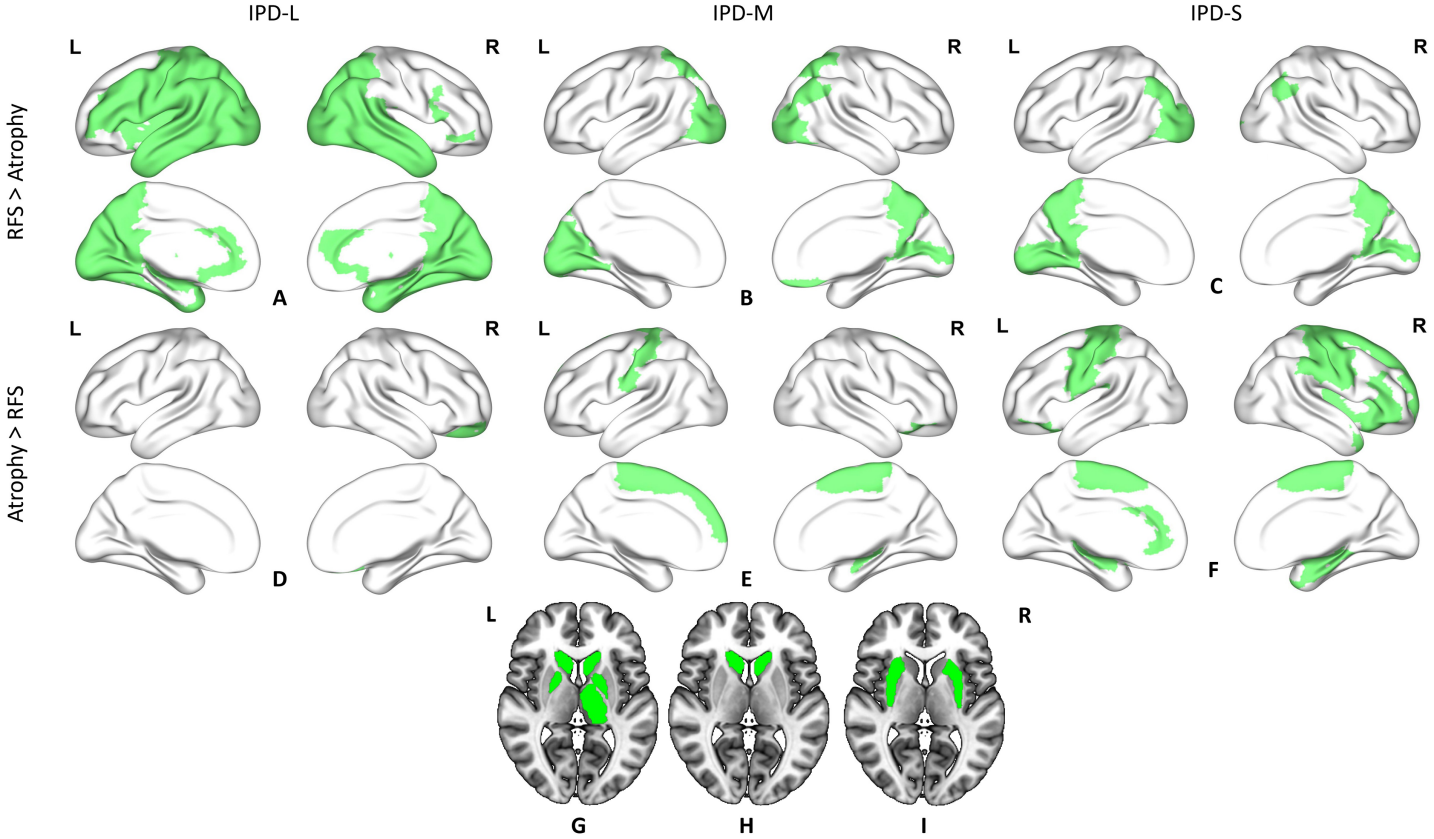
**(A–C)** RFS predominates over atrophy in the cerebral cortex. **(D–F)** Atrophy predominates over RFS in the cerebral cortex. **(G)** RFS predominates over atrophy in the basal ganglia nuclei of IPD-L patients. **(H)** RFS predominates over atrophy in the basal ganglia nuclei of both IPD-M and IPD-S patients. **(I)** Atrophy predominates over RFS in the basal ganglia nuclei of both IPD-M and IPD-S patients. Green indicates structural atrophy or RFS. L, left; R, right.

In early stages, the number of regions where RFS predominated over atrophy (57 regions) substantially exceeded those where atrophy was more severe (1 region). In late stages, the number of regions with more pronounced atrophy than RFS (25 regions) surpassed that of regions where RFS was dominant (12 regions). A comparison between cerebral atrophy and RFS is presented in are presented in Figure 5, with detailed data provided in Supplementary Table S3.

## Discussion

This study utilized integrated PET/MR to systematically evaluate GM atrophy and FDG metabolic changes in patients with IPD. Our findings are fourfold: (I) As disease progressed, the patterns of GM atrophy and RFS in IPD patients expanded from focal brain regions to widespread areas. (II) A characteristic pattern of cerebral hypermetabolism was identified in IPD. (III) H-Y staging showed significant negative correlations with both GM atrophy and RFS in multiple brain regions including the frontal, parietal, temporal, and occipital lobes, limbic system, and basal ganglia, with more substantial correlations observed with structural atrophy. Concurrently, positive correlations were found with hypermetabolism in sensorimotor cortex, globus pallidus and amygdala. (IV) Significant spatial dissociation was observed between GM atrophy and RFS in IPD patients. During mild IPD stages, the spatial extent of RFS considerably exceeded that of atrophy; as disease progressed to moderate–severe stages, atrophic changes gradually expanded and became the more predominant imaging manifestation.

In patients with mild to moderate IPD (H-Y stages 1–2), GM generally shows no significant atrophy (Ibarretxe-Bilbao et al., 2012) or exhibits only limited atrophy confined to a few brain regions (Jia et al., 2015; Guimaraes et al., 2016). As the disease progresses, multiple studies have observed more extensive GM atrophy in various brain areas including the frontal, temporal, parietal, and occipital lobes, as well as subcortical structures (Guimaraes et al., 2016; Lewis et al., 2016; Cui et al., 2020; Laansma et al., 2021; Charisse et al., 2022; Yazdan Panah et al., 2023; Moradi et al., 2024). The findings of this study are consistent with the above reports: GM atrophy in mild to moderate IPD was restricted to the left transverse temporal gyrus, while in severe cases, it further extended to multiple regions such as the frontal, parietal, and temporal lobes, as well as the limbic system. In this study, patients with mild, moderate, and severe IPD all exhibited RFS in visual-related areas of the occipital lobe. Compared to structural changes, the degree of RFS in this region exceeded that of atrophy across all severity groups. Previous studies have also confirmed functional abnormalities (Weil et al., 2016; Nieto-Escamez et al., 2023) and RFS (Zang et al., 2023; Li M. et al., 2025) in visual-related occipital regions in IPD patients. Neuroimaging research further indicates that a significant increase in functional gradient variability within the visual network is closely associated with synchronous decline in FDG metabolism in the visual cortex (Zang et al., 2023). Studies have pointed out that glucose metabolism provides the energy foundation for neural functional network activity, as it primarily supports the energy consumption required for neural communication processes (Hyder et al., 2013). Moreover, in this study, RFS in severe IPD patients further extended to multiple cerebral regions including the frontal, parietal, temporal, and occipital lobes, a result consistent with several previous studies (He et al., 2025; Li S. et al., 2025; Silva-Rodriguez et al., 2025).

In the present study, we observed hypermetabolism in the sensorimotor cortex (including the precentral gyrus, postcentral gyrus and paracentral lobule), and putamen among patients with moderate-to-severe IPD, confirming a characteristic pattern of cerebral hypermetabolism in IPD. This pattern is consistent with previously reported hypermetabolic distributions (putamen, globus pallidus, thalamus, pons, cerebellum, and sensorimotor cortex) and has been supported by literature across different disease stages (Matthews et al., 2018; Liu et al., 2020; Meles et al., 2020; He et al., 2025; Li M. et al., 2025; Li S. et al., 2025). Mechanistically, hyperexcitability of the subthalamic nucleus (STN) enhances inhibitory output to the internal globus pallidus/substantia nigra pars reticulata, leading to dysfunction within the thalamo-cortical circuitry. Concurrently, aberrant STN activity drives hypermetabolism in the lentiform nucleus via neural pathways, which further exacerbates thalamic hypermetabolism (Li M. et al., 2025).

Studies have demonstrated a negative correlation between H-Y staging and metabolic activity in regions such as the frontal, parietal, and temporal lobes, basal ganglia, thalamus, pons, and cerebellum (Huang et al., 2007; He et al., 2025; Li S. et al., 2025). The present study further revealed that higher H-Y stages showed significant positive correlations with both GM atrophy and RFS across multiple brain regions, including the frontal, parietal, temporal, and occipital lobes, limbic system, and basal ganglia. Notably, the correlation between H-Y stage and structural brain atrophy was more pronounced and involved a broader range of regions compared to metabolic changes. Additionally, this study indicated that elevated H-Y staging was also positively correlated with hypermetabolism in areas such as the sensorimotor cortex, amygdala, and globus pallidus. These findings are supported by a longitudinal study that followed patients with mild IPD, conducting FDG metabolic imaging at baseline, 24 months, and 48 months. The results demonstrated that disease progression in IPD was significantly associated with hypermetabolism in the sensorimotor cortex, globus pallidus, pons, and STN (Huang et al., 2007).

Currently, there is considerable heterogeneity in the findings regarding the association between cerebral structural changes and metabolic abnormalities. Gonzalez-Redondo et al. (2014), based on *Z*-Score mapping comparisons, reported that the spatial extent of hypometabolism significantly exceeded that of atrophy in patients with IPD–mild cognitive impairment (IPD-MCI) and IPD with dementia (PDD), particularly within the cerebral cortex. However, the study also identified small cluster-like atrophic changes in regions such as the precentral gyrus, supplementary motor area, temporal lobe, hippocampus, putamen, globus pallidus, and thalamus, where the degree of atrophy even surpassed that of hypometabolism—a phenomenon similarly reported in AD (Salmon et al., 2024), suggesting possible distinct underlying pathological mechanisms (Gonzalez-Redondo et al., 2014). A meta-analysis (Albrecht et al., 2019) integrating two meta-analytic algorithms revealed that hypometabolic regions detected by FDG-PET in IPD patients (e.g., bilateral inferior parietal lobule and left caudate nucleus) were more extensive and consistent than structural atrophy identified by MRI (e.g., the middle occipital gyrus). Through spatial covariance analysis, Liu et al. (2020) compared PET- and MRI-derived Parkinson’s disease-related pattern networks and concluded that both hold promise as diagnostic biomarkers. The results of this study demonstrate a significant dissociation between RFS and GM atrophy, with a moderate correlation observed only in the left inferior parietal lobule and temporal pole of superior temporal gyrus in severe IPD patients. In the early disease stage (mild IPD), the brain regions involved in RFS far exceeded those showing GM atrophy. As the disease progressed to moderate and severe stages, the extent of cerebral atrophy gradually expanded and became a more dominant imaging feature. The discrepancy between the two gradually diminished with disease progression. By the severe IPD stage, some regions—mainly including the frontal and parietal lobes, limbic system, and bilateral putamen—even exhibited more pronounced GM atrophy than RFS. Based on these findings, we propose the following possible mechanisms. Early synaptic dysfunction in Parkinson’s disease primarily results from the abnormal aggregation of *α*-synuclein at presynaptic terminals, which subsequently induces mitochondrial damage and synaptic injury (Di Maio et al., 2016; Ghiglieri et al., 2018; Gcwensa et al., 2021; Calabresi et al., 2023; Su et al., 2026). Neuropathological evidence from Parkinson’s disease patients (Kordower et al., 2013), PET studies (Nandhagopal et al., 2011), and animal model experiments (Phan et al., 2017; Thomsen et al., 2021) consistently indicate that the earliest changes in Parkinson’s disease are axonal terminal dysfunction and loss, which occur much earlier than somatic cell death and the formation of typical Lewy bodies. Numerous studies have confirmed that reduced FDG uptake effectively reflects such synaptic dysfunction (Patel et al., 2005; Magistretti and Allaman, 2015; Andersen et al., 2023; Wang et al., 2025). If synaptic dysfunction persists over a long period, it will further lead to neuronal damage and ultimately manifest as a reduction in grey matter volume (Ghiglieri et al., 2018).

Previous studies have indicated that the volume of the caudate nucleus is significantly reduced in patients with mid-to-late-stage IPD compared to HC, and a declining trend is already observable in early-stage IPD (Lewis et al., 2016; Cui et al., 2020). A longitudinal study further suggested that the progression of PDD is closely associated with hypometabolism in the caudate nucleus (Bohnen et al., 2011). These findings imply that structural and metabolic alterations in the caudate nucleus may serve as biomarkers for early disease progression in IPD, holding potential clinical applicability (Lewis et al., 2016; Sampedro et al., 2019). In the present study, significant GM atrophy in the caudate nucleus was not observed in patients with mild, moderate, or severe IPD; however, moderate and severe IPD groups exhibited RFS in the caudate nucleus. Further comparative analysis revealed that the degree of RFS in the bilateral caudate nucleus exceeded the degree of atrophy across all disease stages of IPD. However, RFS in the caudate nucleus is not unique to IPD, as this phenomenon is also widely observed in other neurodegenerative disorders (Seniaray et al., 2022).

Notably, the present study did not detect significant RFS or GM atrophy in the putamen or globus pallidus, a result consistent with some previous reports (Liu et al., 2020). One possible explanation lies in the compensatory mechanisms of the dopaminergic system: by the time motor symptoms manifest in IPD patients, more than 70% of dopaminergic neurons have already been lost (Gonzalez-Rodriguez et al., 2021). In the early stages of the disease, compensatory mechanisms—such as enhanced dopamine release, increased turnover, and reduced reuptake—may help maintain relatively stable dopamine levels (Heng et al., 2023), potentially preserving the VGM of globus pallidus and putamen (Blesa et al., 2017). Other studies also support this finding, reporting no significant involvement of VGM in the globus pallidus and putamen in moderate IPD patients (Liu et al., 2020). A multicenter study proposed that GM atrophy in the bilateral putamen begins in the moderate IPD stage and worsens further in severe stages (Laansma et al., 2021). Furthermore, the current study observed an increase in VGM of the globus pallidus in moderate and severe IPD patients, a phenomenon also reported in certain previous studies (Jia et al., 2015).

This study directly compared the relative degrees of GM atrophy and RFS using *Z*-scores, while the integrated PET/MR technique circumvented the spatiotemporal mismatching problems of traditional multimodal imaging. Given the relative paucity of studies on the structure-metabolism correlation in IPD, this study has good prospectiveness and novelty.This study has several limitations. First, the analysis was based solely on the H-Y stage as a clinical indicator and did not incorporate cognitive and motor function assessments. The absence of these variables limited the in-depth exploration of comprehensive phenotypical characteristics of the disease and their associations with imaging changes. Additionally, although this study inferred the temporal sequence of pathological changes by comparing patients at different disease stages, it employed a cross-sectional rather than a longitudinal follow-up design. Therefore, the relevant conclusions warrant further validation using longitudinal data.

## Conclusion

This study, utilizing integrated PET/MR analysis, revealed that cerebral GM atrophy and FDG metabolic abnormalities in IPD patients progressively extend from focal to widespread brain areas as the disease advances. A significant spatial dissociation was observed: in early stages, the extent of RFS exceeds that of atrophy, while structural atrophy gradually becomes the dominant manifestation with disease progression. These findings indicate that structural and metabolic alterations in IPD do not evolve synchronously across different stages, potentially reflecting the dynamic evolution of underlying pathological mechanisms.

## Data Availability

The raw data supporting the conclusions of this article will be made available by the authors, without undue reservation.

## Footnotes

1
https://www.fil.ion.ucl.ac.uk/spm/software/spm12/
