# Supplementary material for: Integrated PET/MRI for the evaluation of gray matter atrophy and metabolic changes in idiopathic Parkinson’s disease
Source: Front Aging Neurosci. 2026 Jul 7;18:1757469. doi: 10.3389/fnagi.2026.1757469 (PMC13385225; doi:10.3389/fnagi.2026.1757469)
Supplement: Supplementary file 1 [file Table_1.DOCX]

Supplementary Table S1 Alterations in Brain Structure and Metabolism

| Group | GM Atrophy | GM Hypertrophy | FDG Hypometabolism | FDG Hypermetabolism |
| --- | --- | --- | --- | --- |
| L vs. HC | --- | bilateral SPL, right Cu, IPL | bilateral CF, left LG, right SOG | right SFGorb |
| M vs. HC | left TTG | GP | bilateral TTG, CF, CN; left IFGtri, MTG, LG; right AG, MOG | bilateral PCL, putamen |
| S vs. HC | bilateral STG, MTG, TTG; left FOp, SFGmorb, IPL, STGtp, ACG; right MFG, IFGorb | GP | bilateral PCu, AG, TTG, CF, MOG, CN;left MFG, IFGtri, IPL, STGtp, MTG, IOG, LG; right IFGop, OLF | bilateral PCL, PoCG, right PreCG, left putamen |
| M vs. L | bilateral SFGmorb, MFG, FOp, AG, IPL, STG, MTG, ITG, STGtp, OLF, CF, FG, LG, SOG, PHG, HIP, ACG, MCG, insula; left SFGm, GR, SMG, PoCG, TTG, amygdala; right PCu, MOG, IOG | --- | right IPL, insula, MFG, SFGmorb, GR | bilateral GP, right PCL |
| S vs. L | bilateral PreCG, SFGdl, SFGmorb, MFG, MFGorb, IFGop, IFGtri, IFGorb, GR, AG, SPL, IPL, PoCG, SMG, STG, MTG, ITG, TTG, STGtp, OLF, CF, SOG, MOG, FG, HIP, PHG, insula, ACG, MCG, amygdala; left FOp, SFGm, LG; right SMA, Cu | --- | bilateral IPL, MFG, right SFGm, SFGmorb, GR, TTG | bilateral PCL, right PoCG, PreCG |

L,IPD-L ; M, IPD-M; S, IPD-S; The dash (-) denotes brain regions without statistical significance; ACG, anterior cingulate gyrus; AG, angular gyrus; CF, calcarine fissure; CN, caudate nucleus; Cu, cuneus; FG, fusiform gyrus; FOp, frontal operculum; GR, gyrus rectus; GP, globus pallidus; HIP, hippocampus; IFG, inferior frontal gyrus; IFGop, opercular part of IFG; IFGorb, orbital part of IFG; IFGtri, triangular part of IFG; IPL, inferior parietal lobule; IOG, inferior occipital gyrus; ITG, inferior temporal gyrus; LG, lingual gyrus; MCG, middle cingulate gyrus; MFG, middle frontal gyrus; MFGorb, orbital part of MFG; MOG, middle occipital gyrus; MTG, middle temporal gyrus; MTGtp, temporal pole of MTG; OLF, olfactory cortex; PCG, posterior cingulate gyrus; PCL, paracentral lobule; PCu, precuneus; PHG, parahippocampal gyrus; PoCG, postcentral gyrus; PreCG, precentral gyrus; SFG, superior frontal gyrus; SFGdl, dorsolateral part of SFG; SFGmorb, medial orbital part of SFG; SFGm, medial part of SFG; SFGorb, orbital part of SFG; SMA, supplementary motor area; SMG, supramarginal gyrus; SOG, superior occipital gyrus; SPL, superior parietal lobule; STG, superior temporal gyrus; STGtp, temporal pole of STG; TTG, transverse temporal gyrus

Supplementary Table S2 Correlation of H-Y Staging with Brain Structure and Metabolism

| Group | Correlation with H-Y Staging |
| --- | --- |
| GM Atrophy | frontal lobe: bilateral PreCG, SFGm, SFGorb, SFGmorb, SFGdl, MFG, MFGorb, IFGtri, IFGorb, IFGop, FOp, GR; parietal lobe: bilateral PCu, AG, SMA, PoCG, SPL, IPL, SMG, PCL; temporal lobe: bilateral STG, MTG, ITG, OLF, TTG, STGtp, MTGtp; occipital lobe: bilateral CF, Cu, LG, SOG, MOG, IOG; limbic system: bilateral HIP, PHG, insula, ACG, MCG, amygdala; basal ganglia: bilateral CN, thalamus |
| FDG Hypometabolism | frontal lobe: bilateral SFGmorb, SFGm, SFGorb, MFG, GR; left SFGdl; parietal lobe: bilateral IPL; left AG; right PCu; temporal lobe: right OLF, TTG; limbic system: right MCG; basal ganglia: bilateral CN |
| FDG Hypermetabolism | frontal lobe: right PreCG; parietal lobe: bilateral PCL; right PoCG; limbic system: bilateral amygdala; basal ganglia: bilateral GP |

Abbreviations are listed in the notes to Supplementary Table S1

Supplementary Table S3 Comparison between Cerebral Atrophy and Hypometabolism

| Group | IPD-L | IPD-M | IPD-S |
| --- | --- | --- | --- |
| hypometabolism＞atrophy | frontal lobe: bilateral IFGop; left MFG, IFGtri, FOp, PreCG; right IFGorb; parietal lobe: bilateral PCu, AG, SPL, IPL; left SMG, PoCG; temporal lobe: bilateral STG, MTG, ITG, STGtp, MTGtp, OLF; left TTG; occipital lobe: bilateral SOG, MOG, IOG, LG, FG, Cu, CF; limbic system: bilateral ACG, PCG, HIP, amygdala; left insula; right PHG; basal ganglia: bilateral CN, GP; right thalamus | parietal lobe: bilateral SPL; right PCu, AG, GR; occipital lobe: bilateral CF; left Cu, LG, MOG, IOG; basal ganglia: bilateral CN | parietal lobe: bilateral PCu, AG; occipital lobe: bilateral CF; left LG, MOG, IOG; limbic system: left PCG; basal ganglia: bilateral CN |
|  |  |  |  |
| atrophy＞hypometabolism | right SFGorb | frontal lobe: left SFGm; right MFGorb; parietal lobe: bilateral SMA, PCL; left PoCG; limbic system: right HIP; basal ganglia: bilateral putamen | frontal lobe: bilateral PreCG, MFGorb; left FOp; right SFGdl, IFGtri, IFGorb; parietal lobe: bilateral SMA, PCL, PoCG; right SMG;  temporal lobe: right MTGtp; limbic system: bilateral HIP, amygdala; left ACG; right insula, PHG; basal ganglia: bilateral putamen |

Abbreviations are listed in the notes to Supplementary Table S1
